# A pragmatic parallel cluster-randomized trial to evaluate the implementation and effectiveness of optimized electronic monitors in improving tuberculosis patients’ treatment adherence in China: study protocol

**DOI:** 10.3389/fpubh.2025.1619461

**Published:** 2025-12-15

**Authors:** Liming Yang, Min Su

**Affiliations:** 1 School of Public Management, Inner Mongolia University, Hohhot, China; 2 Department of Fundamental Theory, Ziyang Administrative College, Ziyang, China; 3 Resident Work Team, Gonghe Village, Ziyang, China

**Keywords:** realistic evaluation, tuberculosis patient, treatment adherence, electronic monitors, optimized EM program, CFIR, ERIC, RE-AIM

## Abstract

**Background:**

Treatment non-adherence poses a serious risk to survival and hinders the improvement of tuberculosis (TB) control effectiveness in Inner Mongolia, China. To improve treatment adherence and health outcomes in Inner Mongolia, this study aims to maximize the impact of an electronic monitor and smartphone app (EM program) by developing interventions that optimize the EM program, putting it into practice and evaluating it, and developing scale-up activities of the optimized EM program.

**Methods:**

First, a Consolidated Framework for Implementation Research will be used to assess the implementation of electronic monitors to improve treatment adherence and health outcomes for TB patients in China and identify the facilitators and barriers. Second, we will use the Expert Recommendations for Implementing Change protocol to identify appropriate implementation strategies to optimize the EM program in the Inner Mongolian context. Third, the optimized EM program will be implemented and assessed during a 12-month pragmatic, parallel, cluster-randomized trial in three chosen cities in Inner Mongolia. The treatment adherence of TB patients will be the main result. The secondary outcomes will be TB treatment outcomes as defined by the World Health Organization, including the treatment completion rate, loss to follow-up rate, treatment failure rate, and treatment-related deaths. Based on the RE-AIM framework, the impact of the improved EM program will also be assessed in comparison to standard care for the subsequent secondary outcomes (reach, effectiveness, adoption, implementation, and maintenance).

**Discussion:**

This study will be the first to develop and implement interventions that improve the treatment adherence and health outcomes of TB patients in addition to developing strategic options for the scalability and generalizability of the optimized interventions in remote areas of China and other low- and middle-income countries. All intervention activities will be developed for incorporation into regular TB care, with strong local ownership. Through the trial, we hope to uncover more information about the long-term effects, efficacy, cost-effectiveness, and practicability of our intervention.

**Trial registration:**

ISRCTN15169616. Registered on 29 July 2023.

## Introduction

The second most lethal infectious killer after COVID-19 (above HIV/AIDS) is tuberculosis (TB). One of the most prevalent infectious diseases, TB affects almost one quarter of the world’s population (1, 2) and is the 13th leading cause of death. Despite significant efforts to fight it, TB is still a global and Chinese public health concern. According to a report by the World Health Organization (WHO), 1.6 million people worldwide died from TB in 2022 (1, 2). China has the second-highest TB burden in the world (3). A total of 84,200 new cases of TB were reported in China in 2021, with a 59/100,000 incidence rate (1).

Despite the availability of effective TB medications, treatment non-adherence has been demonstrated (4, 5). This continues to be a major obstacle to improving TB control effectiveness and poses a significant risk to survival (6). Treatment non-adherence is a growing concern given the evidence of its high prevalence, correlation with unfavorable outcomes (such as treatment failure, relapse, and drug resistance), and correlation with rising healthcare costs (6, 7). Due to its low population density, harsh weather, lengthy travel distances, and lack of human resources to support the implementation of directly observed treatment, treatment non-adherence is even worse in Inner Mongolia, China (8). To ensure TB intervention adherence in Inner Mongolia, urgent action is needed.

Electronic medication monitor (EMM) boxes, a type of digital health technology, are a promising tool for enhancing TB patients’ treatment adherence. The EMM box helps TB patients remember to take their medications, but it also alerts health care professionals to potentially dangerous health behavior patterns in time for them to take appropriate action before treatment is interrupted or fails (9). It can be connected to TB patients’ smartphones to provide additional alerts and notify caregivers right away about their medication behavior histories, providing them with prompt support if treatment adherence is interrupted and incorrectly recorded (9–11). This addresses the shortcomings of directly observed treatment, video-observed treatment (VOT), and short messaging service, providing TB patients with more convenient and adaptable visual and audible reminders for taking their medications, as well as more individualized follow-up, prescription, and consultation (12, 13).

Most of the current literature focuses on the benefits, effectiveness, cost-effectiveness, robustness, acceptability, feasibility, and usability of electronic monitors to enhance TB patients’ treatment adherence and health outcomes (14–19). Little information exists on how to determine the best course of action and solutions based on problems that TB patients face. Furthermore, to the best of our knowledge, little research has been performed on implementation strategies to produce data that can be generalized and used to guide future scale-up in remote regions of China and other low- and middle-income countries (LMICs).

Implementation research seeks to understand what functions in actual settings, for whom, and under what conditions (20). Theory-based implementation strategies are becoming more popular. Additionally, there is a growing understanding of the necessity of using framework-based approaches to understand the factors that contribute to the success of a program or intervention (21). Future research on the electronic monitor can be greatly improved by implementation research. In 2021, Inner Mongolia began using an electronic monitor and smartphone app (EM program). Therefore, determining how to maximize the EM program’s impact is crucial to enhance treatment adherence and health outcomes in Inner Mongolia. The EM program should be optimized after the successful initial phase, considering the requirements in response to the barriers and facilitators of implementing electronic monitors in the real-world setting. This study will generate a context-sensitive, evidence-based implementation strategy for this optimization in collaboration with Chinese Center for Disease Control and Prevention (China CDC) and Inner Mongolia CDC, with the goal of enhancing the reach, effectiveness, adoption, implementation, and maintenance of the EM program in Inner Mongolia from a multidisciplinary research perspective.

## Methods and analysis

The Standards for Reporting Implementation Studies guidelines were followed when reporting this protocol (see Supplementary Data Sheet 1 for details) (22).

### Study objectives

By maximizing the EM program’s implementation, the overall objective is to improve the treatment adherence and health outcomes of TB patients. The study’s specific goals include:

1. To identify the barriers and facilitators of implementing EM program that aim to improve TB patients’ treatment adherence and health outcomes in Inner Mongolia, China.
2. To co-produce context-sensitive, and evidence-based interventions that optimize the EM program, and implement the optimized interventions, and evaluate the implementation strategies regarding acceptability, cost-effectiveness, and long-term effects in remote areas of China.
3. To develop strategic options for the scalability and generalizability of the optimized interventions.

### Study design

**Figure 1:**
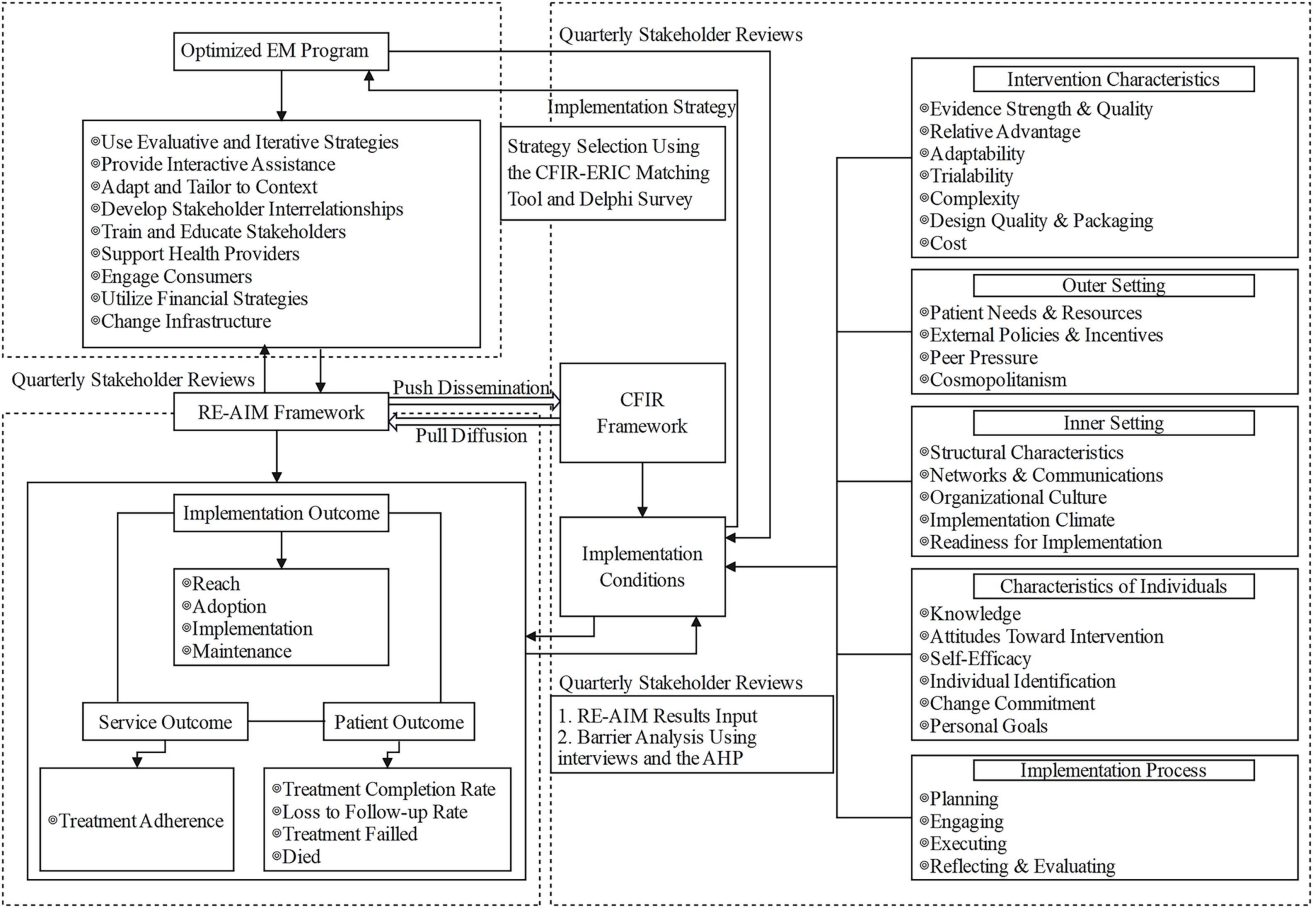
Theoretical model for this study.

The optimized EM program’s implementation and evaluation under the theory of implementation science are synthesized in the study’s theoretical model (Figure 1). To improve TB patients’ treatment adherence and health outcomes in China, the EM program will be implemented. The Consolidated Framework for Implementation Research (CFIR) framework will be used to identify the facilitators and barriers to this (23). The Expert Recommendations for Implementing Change (ERIC) protocol will then be used to determine the best implementation strategies for the EM program in the context of China’s remote regions (24). The implementation strategies will be assessed in terms of implementation outcome, service outcome, and patient outcome using the RE-AIM framework (25). The implementation and efficacy of the optimized EM program will be assessed using these indicators, which include reach, effectiveness, adoption, implementation, and maintenance, based on the theoretical model. Figure 1.

### Project activities

#### Phase 1: CFIR-based implementation determinants

##### Conceptual framework

We will employ a framework-based implementation research insight approach to systematically examine, identify, and synthesize the facilitators and barriers to implementing EM programs that are meant to increase treatment adherence and health outcomes in TB patients. One of the most widely used and operationalized frameworks for predicting or explaining implementation effectiveness barriers and facilitators is the CFIR (26). Intervention characteristics, outer setting, inner setting, individual characteristics, and process are the five main domains of CFIR, made up of 39 sub-domains. These 39 sub-domains affect the implementation process and outcomes. This framework creates a structured and methodical way to evaluate the environment in which implementation takes place by integrating constructs from published theories and models (27). It might serve as a good starting point for developing multifaceted implementation strategies to increase the uptake, usefulness, and longevity of electronic monitors (28).

##### Study participants

The three groups of participants will be (1) EM program managers, coordinators, and staff; (2) community health center and township leaders and public health staff; and (3) patients and their family supporters during treatment.

##### Data collection and analysis

A semi-structured interview will be used to gather the data. To create the interview guide and direct data collection, we will use the CFIR. Each sampling site will host in-depth interviews. We will consult the managers and staff at each site to determine which employees are best suited for an interview. We will obtain informed consent forms. At each site, interviews will take place over the phone or in person and last for 60 to 90 min. We will take notes and record the interviews. Using a codebook available on the CFIR website (29), two researchers will independently code the recordings and notes from the first three sites to the CFIR framework’s constructs. To prioritize the 39 sub-domains, we will conduct an Analytic Hierarchy Process (AHP) with 10 local implementers. Weight allocation is based on expert consensus through the Delphi method, supported by the following theoretical and practical considerations: (1) Modifiability - Theoretical basis: Following the “modifiability principle” in implementation science, we prioritize intervention points with the highest cost-effectiveness. Practical significance: In resource-limited rural areas, low-cost improvement measures are more sustainable for promotion. (2) Impact – Theoretical basis: Adhering to the “maximum impact principle” to concentrate resources on addressing the most significant barriers to treatment adherence. (3) Resource Intensity – Theoretical basis: Grounded in “resource adaptation theory,” considering the actual implementation capacity of the Inner Mongolia region. Practical significance: Ensuring implementation strategies match local health resource allocation. (4) Time Sensitivity – Theoretical basis: Based on “urgency stratification” to identify implementation barriers requiring immediate attention. Participants will perform pairwise comparisons of sub-domains to construct judgment matrices. Eigenvector methods will derive final weights, with consistency ratios < 0.1 serving as a statistical verification tool to ensure logical consistency of judgments, while the weight allocation itself is founded upon in-depth analysis of implementation science theories and local practical conditions. Expert consensus will be developed through a two-round Delphi process: Round 1: Ten local implementers (5 CDC staff and 5 clinicians) independently ranked the importance of each criterion; Round 2‌: The expert panel will discuss and reach a consensus, specifically considering: (1) The geographical particularities of Inner Mongolia (e.g., low population density, pastoral distribution); (2) The specific needs of TB patients (e.g., low literacy rates, linguistic diversity); (3) The carrying capacity of the existing health system. To validate the rationality of weight allocation, we will employ the following methods: (1) Content Validity Test: Three implementation science experts will evaluate the weight allocation scheme for theoretical soundness and practical relevance; (2) Practical Relevance Verification: The weight allocation results will be compared with the practical work experience of Inner Mongolia CDC to ensure alignment with local implementation realities; (3) Sensitivity Analysis: Strategy selection robustness will be tested under different weight combinations to assess the stability of prioritization outcomes.

Results will inform Tier 1 (top 20% weighted) determinants for ERIC strategy matching. Additionally, two researchers will conduct the analysis independently at each stage of coding, indexing, and interpreting and come to a consensus in the event of any disagreement (30). The policy documents, institution reports, and project meeting notes will also be used throughout the study to identify implementation challenges and opportunities, improve the program, and give site staff feedback (31). The qualitative analysis program NVivo 12.0 will be used to examine all interview data. The narrative synthesis of the general characteristics of the included participants will be tabulated and conducted following the findings of the qualitative analysis, and the five domains of the CFIR framework will be used to summarize the common implementation barriers and facilitators of implementing the EM program. Thematic findings from cases with Cohen’s kappa (*κ*) ≤ 0.6 will be analyzed separately to inform Phase 3 RE-AIM evaluation.

#### Phase 2: ERIC-based implementation strategies

We will code the interview and then determine the most effective implementation strategies to address the contextual barriers discovered in phase 1, using a cutting-edge implementation science approach based on the ERIC protocol (32). To contextualize generic CFIR-ERIC recommendations, we will conduct a 2-round Delphi survey with 7 county CDC staff (≥ 5 years’ experience in TB control) and 7 patient representatives, including 4 urban patients (2 low-literacy older adults, 2 young Chinese-speaking patients) and 3 pastoral patients (2 Mongolian-speaking nomads, 1 Chinese-speaking settler). In Round 1, panelists will independently rate all ERIC strategies on feasibility (1–5 Likert) given local geography/climate, cultural appropriateness for nomadic lifestyles, and digital literacy demands. Round 1 ratings will incorporate AHP-derived weights to stratify strategies (e.g., prioritizing high-modifiability/high-impact combinations). Round 2 will involve a consensus meeting to finalize strategies, with *κ*-statistic calculated for inter-panel agreement on strategy retention/drop decisions (*κ* threshold > 0.6) and priority adaptations (e.g., Mongolian audio substitutes for text alerts). We will use the CFIR-ERIC Implementation Strategy Matching Tool v0.53^1^ to choose strategies based on CFIR barriers. The CFIR-ERIC Matching Tool was developed with assistance from implementation experts (*n* = 169) who responded to the survey and chose up to seven implementation strategies they believed would best address each CFIR barrier (33–35). The top row of an output table lists the seven CFIR barriers (condensed to only include solutions with the highest level of support). In the first column is a list of ERIC implementation strategies. The order of the strategies is based on their overall level of support. The phrase “cumulative percent” denotes the degree to which that strategy is supported by all seven CFIR barriers (33). To create more detailed recommendations, we will combine the results of the CFIR-ERIC tool with advice given to us during interviews and found in the literature (32). We will also combine these recommendations with the local context in this study.

#### Phase 3: Implementation and evaluation of optimized EM program

##### Study design

Constrained randomization of clusters to arms will be used in this 12-month pragmatic parallel cluster-randomized trial study (36–38). Community health centers and township hospitals make up the randomization unit. Clusters will be randomly assigned in a 1:1 ratio with constrained randomization to enforce a 20-km buffer zone between intervention and control clusters, excluding registered nomadic herders via EMM box GPS tracking during transhumance. Real-time nomadic mobility alerts will be triggered via National ID-based tracking for non-exempt patients exceeding 20-km movement, with trajectory validation. The optimized strategies will be prepared at all intervention sites over a 3-month implementation and washout phase (39). The optimized EM program will be fully implemented and the intervention arm will start at the same time at all sites. The optimized EM strategies will not be used in the control arm’s standard of care.

##### Setting

To represent the east, central, and western regions of Inner Mongolia, China, the study will be carried out in the cities of Chifeng, Hohhot, and Baotou. We chose these study locations based on the extent to which the EM program has been implemented, the region, the size of the population, the traffic situation, and the program’s representativeness. With a population of 4.04 million and a land area of more than 9,000 km^2^, Chifeng is located in the east of Inner Mongolia. In 2021, its per capita gross regional product (GRP) was RMB 49,069. Hohhot, the capital of Inner Mongolia, is in the region’s center. It has a population of 3.50 million and a GRP of RMB 53,026 as of 2021. With a population of 2.72 million and a land area of over 27,768 km^2^, Baotou is located in the west and had a GRP of RMB 53,026 in 2021.

##### Participants

The study sample will include patients diagnosed with TB in dispensaries following national and international care guidelines (8), who are initiating a standard 6-month chemotherapy regimen at community health centers or township hospitals. Eligible patients must meet the following criteria: aged ≥ 15 years; no speech, mental, visual, auditory, or other communication impairments; ‌and no cohabitating immediate family members (parents, spouse, children) currently enrolled in this trial.‌ Other cohabitation relationships (e.g., friends, distant relatives, roommates) are not excluded.

Immediate family members are defined as parents, spouse, and children. Other cohabitation relationships (e.g., friends, distant relatives, roommates) are not excluded and will be included in sensitivity analyses to assess environmental influences on treatment outcomes.

##### Intervention

**Figure 2:**
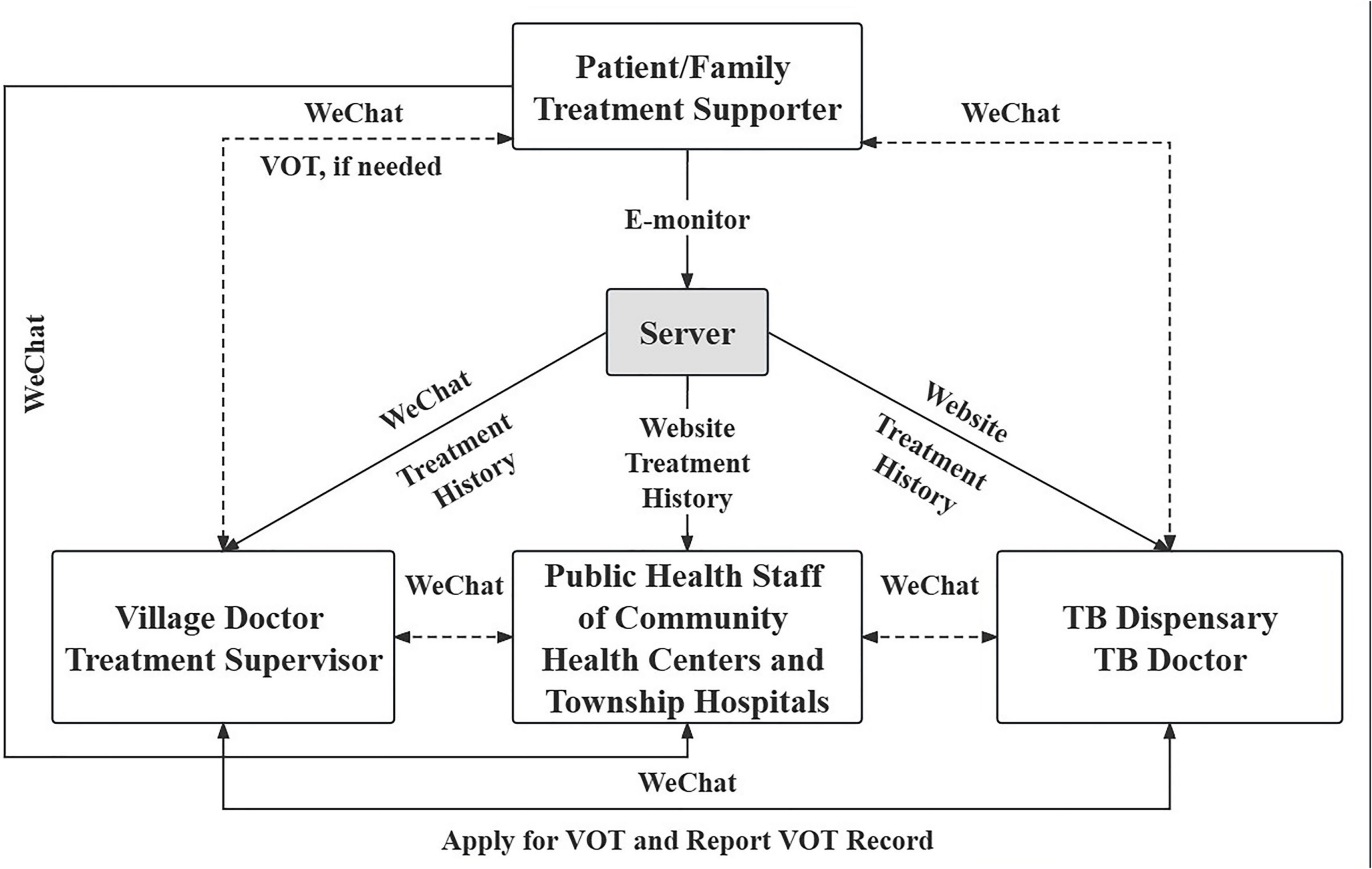
The operation mode of EM program.

Primary care facilities (such as community health centers and township hospitals) will use the intervention. Facilities that are part of the intervention will run the optimized EM program, and those that are not will run the standard EM program. Community health centers and township hospitals had to sign up for the electronic monitors in accordance with the current EM program. Beyond holding the TB medication, the electronic monitors served two purposes. First, they used human voice recordings to remind patients to take their medication on time. Second, they linked a computer with a mobile app to send the history of patient adherence to a cloud-based server (See Figure 2 (8) for details of the operation mode of EM program) (8). The optimized program incorporates three technical isolation measures: (1) Dynamic QR codes (V1-V40 rotation) with geofencing to block cross-cluster device activation; (2) Mandatory bilingual (Mandarin/Mongolian) confidentiality pop-ups triggered upon EMM access‌, combined with monthly WeChat audits via Tencent Cloud OCR scans to detect shared interfaces and with enforced tiered responses from warnings to account suspension; and (3) Cultural isolation through Mongolian-language interfaces and audio prompts blocking operational replication by non-assigned patients. We will incorporate ERIC-based implementation strategies into the current EM program to create an optimized EM program. Figure 2.

To address literacy and linguistic barriers in Inner Mongolia, the optimized system incorporates three key adaptations: (1) Pictorial quick-reference cards illustrating device activation, QR code scanning, medication intake confirmation, battery charging, and troubleshooting procedures; (2) Mongolian audio prompts for dose reminders, low-battery alerts, and operational error feedback; (3) Bilingual interface allowing real-time switching between Mandarin Chinese and Mongolian, with icon-based navigation for low-literacy users. These adaptations complement the existing WeChat-based family support system while maintaining all original technical functions of cloud-based adherence monitoring.

##### Outcomes

The primary study hypothesis is that the optimized EM program will result in higher rates of treatment adherence than standard care. As a result, for TB patients, treatment adherence will be the main result. Treatment adherence will be calculated as the percentage of prescribed doses actually taken by the patient each month. The formula is: ‌Treatment Adherence (%) = (Number of doses actually taken / Number of prescribed doses) × 100%‌. For example, if a patient is prescribed 30 doses per month and actually takes 25 doses, their treatment adherence would be (25/30) × 100% = 83.3%. Adherence will be dichotomized as high adherence (≥80% of doses taken) or non-adherence (<80%). The secondary outcomes for TB treatment will be (1) the treatment completion rate, (2) loss to follow-up rate, (3) treatment failure, and (4) death, as defined by the WHO standard definitions (8, 40). Based on the RE-AIM framework, the impact of the optimized EM program will also be assessed in comparison to standard care for the subsequent secondary outcomes (reach, effectiveness, adoption, implementation, and maintenance) (41, 42). For cases with *κ* ≤ 0.6, Phase 1 qualitative findings will inform the “implementation” and “maintenance” RE-AIM domains to address adherence barriers and enhance sustainability. Table 1 provides additional outcome information. Table 1.

**Table 1:** Outcomes, indicators, and indicator definition by RE-AIM framework.

Outcome	Indicator	Indicator definition
Service outcome	Treatment adherence	Percentage of prescribed doses actually taken each month
Patient outcome	Treatment completion rate	A TB patient who finished treatment without having evidence of treatment failure, but who did not have negative sputum smear or culture results in at least one prior instance or in the final month of treatment, either because the tests were not conducted or the results were not available
	Loss to follow-up rate	A TB patient who did not initiate treatment or whose treatment was interrupted for two consecutive months or more
	Treatment failed	A TB patient who received treatment and had positive results from a sputum smear or culture at month five or later
	Died	A TB patient who passed away before starting treatment or while receiving it, for any reason
Implementation outcome	Reach	Patient-level coverage among all eligible incident TB cases in the catchment area
	Effectiveness	The effects of the improved e-monitoring program on TB treatment outcomes, treatment adherence, and financial outcomes
	Adoption	The proportion of primary-care facilities that formally integrate the optimised EM into routine workflow
	Implementation	Fidelity: extent to which the optimized e-monitoring program is implemented as per the original protocol
		Feasibility: extent to which the optimized e-monitoring program can be implemented in a specific setting
		Outer context: macro-level external factors including social, funding, and leadership
		Inner context: micro-level internal factors including CDC partnership, programmatic staff, feedback, primary care facility, and individual level
	Maintenance	Sustainability of effectiveness: views on maintaining effectiveness from policymakers, directors of CDC and primary care facilities, healthcare providers, and TB patients
		Stakeholders’ satisfaction: satisfaction with the effectiveness and implementation strategy of the optimized e-monitoring program among policymakers, directors of CDC and primary care facilities, health care providers, and TB patients
		Financial sustainability: views on funding and return on investment from policymakers and directors of CDC and primary care facilities
		Institutionalization of interventions: core components that are transferable and require local adaptation for replication in other settings

##### Randomization and blinding

After recruitment, randomization will be conducted using the computer random list generated by STATA 15.0 software. A 1:1 ratio of patients will be randomly assigned to the intervention group or the control group. Sealed envelopes containing the group numbers will be kept by the research manager, who is not directly involved in participant recruitment or follow-up. The research designer will keep the group assignment results until the end of the data analysis period (43, 44).

##### Sample size

Because our sample size is insufficiently large, we used simulation techniques to examine long-term results. Our calculations used a 40% control group proportion and were based on people who did not follow through with monthly treatments (8, 14). To study the impact while maintaining equal distributions among the treatment groups, we created six outcomes for patients in the simulation. The success rate for the control group was 0.4, whereas the success rates for the intervention group varied. The results were generated at random with a fixed success rate. All patients’ monthly outcomes showed a dependable degree of correlation. We produced these simulated result data using a program called “simstudy” (45). Even though our simulations indicated non-conservative effect estimates, we decided to stay with the original correlation structure. Based on the data, we developed a mathematical model using a different software program called “geepack” that allowed us to calculate the precise differences in outcomes between the treatment groups (46). This model had variables for the month and the treatment group. For each group hypothesis, we extracted data pertinent to the predicted treatment effects and repeated this process 1,000 times, calculating power based on the proportion of data 0.05. Since sources of missing data can still contribute to all of their results, we assumed no missing data. To detect a 12-percentage-point improvement in the outcomes of the intervention group with 81.5% certainty, we would need 300 patients, assuming a non-adherence rate of 40% per month in the control group and a correlation of 0.5 among patients. The Monte Carlo simulation for *κ* analysis will adopt the same correlation structure (*ρ* = 0.5) as the power calculation.

##### Pilot stage

To determine whether the intervention and the trial can be implemented, a political study will be carried out. To test the randomization and recruitment procedures, 30 new pulmonary TB patients – 10 in each district (Chifeng, Hohhot, and Baotou) meeting either an education level ≤ primary school or Mongolian monolingual status – will be recruited and randomized into the intervention or control arm as pilot cases. The feasibility and acceptability of the intervention will then be evaluated after 8 weeks of observation.

During this period, cultural adaptation needs will be assessed. Bilingual (Mandarin/Mongolian) System Usability Scale (SUS) assessments will be conducted at device handover (T0) and after a 2-week adaptation period (T1). Iterative refinements will be implemented through three cycles (2 weeks/cycle), targeting SUS scores ≥ 70. Modifications will focus on pictorial interfaces, audio instructions, and workflow simplification, with all adjustments requiring *κ* > 0.8 inter-rater reliability for problem identification. The implementation plan will be revised based on the pilot study results.

### Societal perspective cost-utility analysis

This economic evaluation adopts a societal perspective incorporating three key cost components: (1) Direct medical costs including EMM procurement and maintenance (with cloud service fees), healthcare personnel training differentiated by urban/rural rates, and outpatient consultations adjusted according to Inner Mongolia’s tiered pricing system; (2) Patient-incurred costs comprising travel expenses calculated as round-trip distance × local transit fare, pastoralists’ fuel costs for private vehicles, and lost herding income quantified via sheep unit equivalents/day with seasonal grazing adjustments; (3) Societal benefits such as averted MDR-TB costs projected over 10 years using WHO-CHOICE unit costs (including indirect costs from transmission interruption), productivity gains measured through the human capital approach applied to employment data, and Quality-Adjusted Life Years derived from EQ-5D-5L surveys at baseline/6/12 months using Chinese health status preference weights. Analytical approaches will report incremental cost-effectiveness ratios for both adherence improvement (ICER) and quality-adjusted life years (ICUR). Sensitivity analyses include discount rate variations (base case 3%, range 0–5%), time horizon scenarios (5/10/20-year projections), probabilistic sensitivity analysis with 10,000 Monte Carlo iterations, and geographic cluster subgroup analyses (Chifeng/Hohhot/Baotou clusters), with all costs standardized to 2025 RMB using China’s CPI health component.

The human capital approach was selected over the friction cost method for productivity loss estimation based on the following considerations: (1) Methodological Appropriateness‌: For chronic conditions like TB requiring long-term therapy (standard 6-month regimen), the human capital approach more comprehensively captures the prolonged productivity losses. This is particularly critical for pastoral patients, since treatment interruption may cause cumulative income impacts spanning several years due to the unique demands of nomadic livelihoods. (2) Research Objective Alignment: As this study aims to assess the comprehensive impact of optimized interventions on patients’ adherence and quality of life, the human capital method better reflects the long-term economic benefits arising from improved treatment compliance. (3) Regional Specificity: In Inner Mongolia’s pastoral regions, TB patients’ mobile lifestyles imply that treatment interruptions can result in sustained negative livelihood impacts over extended periods, which the human capital approach can effectively quantify. (4) Data Feasibility: Friction time, vacancy period, and other key parameters required for the friction cost method are lacking for Inner Mongolia, while employment and wage data essential for the human capital approach are more accessible and verifiable through local statistical sources. (5) International Standards Compatibility: The WHO recommends the human capital approach for economic evaluations in TB, facilitating international comparison of findings and policy implications. (6) Policy Relevance: From a public health decision-making perspective, estimates derived from the human capital approach more accurately reflect the true economic burden of TB on individuals and society in the Inner Mongolian context.

Consequently,‌ to calculate productivity losses ‌using this approach‌, we will source employment data and wage rates for Chifeng, Hohhot, and Baotou from the 2025 data ‌published‌ by the local Bureau of Statistics and the ‌Human Resources and Social Security‌ departments.

### Follow-up study

We will enhance the study’s rigor by systematically tracking participants using their national ID numbers within Inner Mongolia’s NTP reporting system. Excluded participants (*κ* ≤ 0.6) will receive separate bias analysis based on baseline characteristics. Starting post-treatment, this process will capture various outcomes, including potential relapses and even cases of loss to follow-up. We’ll monitor relapse cases through the national TB reporting systems for 24 months after treatment completion. Recognizing the possibility of participants relocating to provinces where national ID reporting for TB cases is not mandatory, we’ll address this gap by conducting a concise survey via treatment supervisors at the study’s conclusion. This survey will gather TB status and treatment location information. Mortality data, requiring national ID inputs, will be collected from China’s vital registration system. The primary focus lies on TB relapse, with mortality as a secondary aspect. Analytical methods mirroring the main trial indicators will be used to compare outcomes between intervention and control groups. In cases of missing follow-up data, a complete-case analysis will be performed and documented separately.

Economic data collection will be enhanced through patient cost diaries recording monthly travel frequency/costs (verified against public transport tariffs) and income loss days (cross-checked with local employment records). Herding income impacts will be assessed using baseline livestock holdings from household surveys and seasonal price fluctuations from regional commodity markets.

### Ethical approval

The Xi’an Jiaotong University Health Science Center’s Ethics Committee granted ethics approval (approval number: 2020–119).

### Data collection

Inner Mongolia CDC will regularly gather patient data, such as name, age, gender, education, occupation, diagnosis, and treatment results. The results of sputum smears performed at the fifth and sixth months, as well as information on conversion at the second month, loss to follow-up, and death, will also be recorded as routinely tracked treatment outcomes (8). The treatment adherence data, collected by the electronic monitors and downloaded from the cloud-based database, will be normalized as percentages of prescribed doses to enable standardized comparisons across patients with varying prescription regimens. To ensure that all missed doses are accurately recorded, we will compare the adherence history obtained from e-monitoring devices with pill counts manually collected at the conclusion of each outpatient visit. Cases with *κ* ≤ 0.6 (indicating device-pill count discrepancies) will trigger qualitative interviews to identify root causes (e.g., device tampering, operational errors, non-adherence), and will be analyzed thematically via NVivo 12.0 per Landis and Koch criteria. Adherence data quality will be further ensured through triple validation‌: (1) Monthly unannounced pill counts (10% participants stratified proportionally across Chifeng, Hohhot, and Baotou populations) with a validity threshold of *κ* > 0.8 (SPSS 28.0); (2) Anti-tampering protocols: Randomized audits + 20 × digital microscopy for blister integrity; (3) Tiered corrections: Device recalibration if 0.6 < *κ* ≤ 0.8; data exclusion followed by root-cause analysis if *κ* ≤ 0.6. While cases with *κ* ≤ 0.6 will undergo root-cause analysis and qualitative interviews to identify potential biases, these cases will remain in the primary Intention-to-treat (ITT) analysis with appropriate statistical handling. Finally, data on implementation costs, such as the number of trainees, the trainers’ labor and travel costs, and the price of the EMM boxes and training materials, will be gathered using the researchers’ work logs. We will implement double entry and examine a representative sample to ensure data entry accuracy.

While stratified random sampling improves validity, two methodological considerations emerge: (1) Potential underrepresentation of mobile populations, adjusted via entropy-weighting with approved anonymized mobility data; (2) Monthly audits could miss short-term adherence variations, mitigated by randomized timing and blister pack integrity checks. The *κ* thresholds follow Landis and Koch but require local context interpretation.

### Statistical analysis

ITT analysis will be performed for the primary analysis, including all randomized participants regardless of adherence data quality or device-pill count discrepancies.‌ For cases with *κ* ≤ 0.6 adherence discrepancies, a three-tier multiple imputation strategy will be implemented to maintain the ITT principle.

The Enhanced Multiple Imputation Procedure for *κ* ≤ 0.6 Cases includes: (1) Level 1 (Baseline and Treatment Patterns) - initial imputation based on observed baseline characteristics (age, gender, education) and treatment assignment status (as fixed covariate); (2) Level 2 (Qualitative Insights) – contextualized imputation incorporating key barriers identified in qualitative interviews (e.g., device tampering, operational errors); and (3) Level 3 (Device and Location Factors) - population-specific imputation considering device types and residence classification (urban/rural). The multiple imputation procedure will use Multiple Imputation by Chained Equations with 20 imputed datasets, using baseline characteristics, treatment assignment status, time variables, and observed adherence patterns. The following variables will undergo imputation: age, gender, education, urban/rural residence type, and monthly adherence rates. Final effect estimates will be obtained by combining results across all imputed datasets using Rubin’s rules.

The Sensitivity Analysis Framework comprises: (1) Primary analysis - ITT analysis including all randomized participants with multiple imputation for missing data; (2) Sensitivity analysis 1 – Complete-case analysis including only cases with *κ* > 0.6 to assess whether the primary ITT results are consistent in the subset of high-quality data (*κ* > 0.6), providing a baseline for comparison with the imputed results for cases with *κ* ≤ 0.6‌; (3) Sensitivity analysis 2 – Household-pairs analysis – This analysis examines adherence trajectories using shared random intercept models, specifically for cohabitating patient pairs (non-immediate family members) with device-recorded versus pill-count adherence agreement *κ* > 0.8; (4) Sensitivity analysis 3 – Bayesian analysis using Markov chain Monte Carlo methods with appropriate prior specifications to evaluate the influence of prior distribution choices on posterior effect estimates‌; and (5) Sensitivity analysis 4 - Bootstrapped confidence intervals (1,000 iterations) for cases with *κ* ≤ 0.6 adherence discrepancies to validate the robustness of the multiple imputation approach under different statistical paradigms.

Covariate-adjusted analyses‌ will be used for all outcomes, with individual consideration of participant clustering in community health centers and township hospitals. Treatment adherence will be analyzed as a continuous variable (percentage) while maintaining the predefined categorization threshold of ≥ 80% for high adherence versus < 80% for non-adherence. Intervention-control group differences will be compared using *t-*tests and $\chi^{2}$ tests (20). Intervention effects will be examined using generalized estimating equation (GEE) models incorporating spatial autocorrelation terms to quantify contamination effects, with sensitivity analyses comparing: (1) Household-clustered GEE models using household IDs as clustering variables with exchangeable correlation structure, adopting an intraclass correlation coefficient threshold < 0.1 based on Inner Mongolia’s low population density (20.19 persons/km^2^) and *κ* weak consistency standards (0–0.2); and (2) Bootstrapped confidence intervals (1,000 iterations) for cases with *κ* ≤ 0.6 adherence discrepancies (8).

Cases with *κ* ≤ 0.6 will be evaluated via bootstrapped confidence intervals (1,000 iterations) and qualitative contextualization. These cases remain included in the primary ITT analysis. For continuous outcomes, mean differences and standardized mean differences will be used to assess intervention effectiveness. For binary outcomes, odds ratios and risk differences will be calculated. Additionally, a mixed-effects model that allows health facility-level estimates to be random effects and accounts for facility- and time-level clustering will be used to compare pre- and post-intervention proportions for reach and adoption (20). Implementation and maintenance will be evaluated using a thematic analysis methodology, to identify emerging themes. The data will be translated, transcribed, verified‌, cleaned, and then analyzed using NVivo 12.

### Trial management

Professor Min Su from Inner Mongolia University and Professor Xiaolin Wei from the University of Toronto will serve as co-guarantors of the trial. The trial dataset will be completely accessible to each of them. To protect the security and privacy of the patients involved, as well as to ensure that all data are collected following ethical standards and only used for research, a data management committee led by outside members will be established. The steering committee for the externally led trial will be made up of representatives from China CDC, Inner Mongolia CDC, and other significant trial team members. It will gather every year. Significant protocol changes will be discussed during the meetings (8).

## Discussion

### Strengths and limitations

The WHO recommends using an electronic monitor, which is recognized as an evidence-based intervention (9). This has received much use in primary healthcare facilities and is regarded as the tool with the best chance of increasing TB treatment adherence (47). Health policymakers and researchers must urgently determine how to maximize the positive impact of the electronic monitor on TB patients’ treatment adherence and negative outcomes. In addition to developing strategic options for the scalability and generalizability of the optimized interventions in remote areas of China and other LMICs, this study will be the first to develop and implement interventions that improve treatment adherence and health outcomes for TB patients. The study has several main contributions. Firstly, a CFIR framework will be used to pinpoint the difficulties and opportunities associated with putting electronic monitors in place, as well as potential areas for intervention alterations and improvements. To optimize the EM program in the context of China’s remote regions, we will secondly use the ERIC protocol to identify appropriate implementation strategies. Thirdly, a 12-month pragmatic, parallel, cluster-randomized trial will be used to evaluate the implementation strategies of the optimized EM program using the RE-AIM framework. An electronic monitor implementation strategy will be developed as a result of this study, which is crucial for those who are scaling up activities in remote areas of China and other LMICs.

However, this study confronts five challenges. First, economic analyses may systematically underestimate three components: (1) informal caregiving burdens; (2) TB transmission externalities; and (3) intergenerational educational impacts. Second, despite employing gold-standard cluster-randomized controlled trials (20) with 20-km contamination buffers, residual risks persist through two primary pathways: (1) offline medication-sharing networks; (2) transient mobility events below the 20-km detection threshold. ‌Notably, low population density (20.19/km^2^ in Inner Mongolia) may mitigate contamination risks from such mobility events. Third, confounding factors, bias, and temporal trends could limit the validity of the findings. Fourth, while AHP-optimized strategy prioritization enhances rigor, the weight allocation might reflect evaluators’ professional biases. Furthermore, although many TB patients in Inner Mongolia lack literacy, they can still use smartphones and WeChat (48), because (1) contacts in WeChat can be identified by icons and (2) WeChat enables smartphones to send audio and video recordings rather than just text. A family member with strong literacy will also be used to teach the TB patient how to use WeChat.

### Trial status and timelines

The trial was registered at Current Controlled Trials: July 29, 2023 (ISRCTN15169616). We started to recruit patients from January 11, 2023. At the original submission of the protocol, we have completed our pilot studies.

This study will be done over a 28-month period, with a 3-month preparation and pilot phase, a 15-month participate recruitment phase, with a 6/7-momth treatment phase for all patients, and a 3-month data analysis and write-up stage (8). A 24-month follow-up study of all participates will be conducted after they end treatment under the optimized EM program to look at the treatment outcomes, and implementation strategies between the treatment arms. Results of the follow-up study will be reported in a separate paper from the trial results. See Table 2 for details of the trial timeline. Table 2.

**Table 2:** trial timeline

**Phase**	**Tasks**	**Study period (month)**																												
		**1**	**2**	**3**	**4**	**5**	**6**	**7**	**8**	**9**	**10**	**11**	**12**	**13**	**14**	**15**	**16**	**17**	**18**	**19**	**20**	**21**	**22**	**23**	**24**	**25**	**26**	**27**	**28**	**29-45**
																												
Preparation	Study design																													
	Pilot and modify design																													
Interventions	Enrolment																													
	Interventions																													
	Control																													
Assessments	Treatment adherence																													
	Treatment outcome data																													
	Societal economic evaluation																													
	Process evaluation																													
	Data analysis and paper draft																													
Follow-up	Long term follow-up																													

#### Informed consent process for low-literacy and monolingual participants

The informed consent process for participants with limited literacy skills or who are Mongolian monolingual speakers includes the following measures:

1. Language-adapted documentation: Bilingual informed consent forms in both Mandarin Chinese and Mongolian; and Inclusion of visual icons and simplified text in all consent materials;
2. Multistep understanding verification: Step 1 - Initial explanation by trained research staff using standardized scripts; Step 2 - Participant recall using the “teach-back” method, where participants restate key concepts in their own words; and Step 3 - Standardized assessment checklist covering: Purpose and duration of the trial; Rights and responsibilities of participants; Potential risks and benefits; Voluntary nature of participation and right to withdraw;
3. Comprehensive support mechanisms: Availability of fluent Mongolian-speaking interpreters during all consent procedures; Audio-recorded consent explanations in both Mandarin and Mongolian dialects; Participant option to involve a literate family member or friend as a witness;
4. Quality assurance protocols: Audio/video recording of consent sessions (with participant permission); Monthly audits of consent documentation quality; Mandatory retraining of research staff if participant understanding assessments fall below 80% proficiency;
5. Ongoing consent maintenance: Regular participant check-ins using culturally appropriate communication methods; Dedicated Mongolian-language helpline for questions and concerns.

Through this process, all enrolled TB patients provided written informed consent for research use of collected data.

## Footnotes

1
https://cfirguide.org/choosing-strategies/
